# Phosphoglycerate mutase, 2,3-bisphosphoglycerate phosphatase, creatine kinase and enolase activity and isoenzymes in breast carcinoma

**DOI:** 10.1054/bjoc.1999.0871

**Published:** 1999-12-08

**Authors:** N Durany, J Joseph, O M Jimenez, F Climent, P L Fernández, F Rivera, J Carreras

**Affiliations:** Unit of Biochemistry, IDIBAPS, Faculty of Medicine, University of Barcelona, Casanova 143, Barcelona, 08036, Spain; Pathological Anatomy Department, Hormonal Laboratory, IDIBAPS, Hospital Cliníc i Provincial, Villarroel 170, Barcelona, 08036, Spain

**Keywords:** 2,3-bisphosphoglycerate phosphatase, creatine kinase, enolase, phosphoglycerate mutase activity and isoenzymes, breast carcinoma

## Abstract

We have compared the levels of phosphoglycerate mutase (EC 5.4.2.1), 2,3-bisphosphoglycerate phosphatase (EC 3.1.3.13), creatine kinase (EC 2.7.3.2) and enolase (EC 4.2.1.11) activities and the distribution of their isoenzymes in normal breast tissue and in breast carcinoma. Tumour tissue had higher phosphoglycerate mutase and enolase activity than normal tissue. Creatine kinase activity was higher in seven out of 12 tumours. In contrast 2,3-bisphosphoglycerate phosphatase activity was lower. Phosphoglycerate mutase, enolase and 2,3-bisphosphoglycerate phosphatase presented greater changes in the oestrogen receptor-negative/progesterone receptor-negative breast carcinomas than in the steroid receptor-positive tumours. Determined by electrophoresis, type BB phosphoglycerate mutase, type BB creatine kinase and αα-enolase were the major isoenzymes detected in normal breast tissue. Types αγ and γγ enolase, types MB and MM phosphoglycerate mutase were detected in much lower proportions. In tumours a decrease of phosphoglycerate mutase isoenzymes possessing M-type subunit and some increase of enolase isoenzymes possessing γ-type subunit was observed. No detectable change was observed in the creatine kinase phenotype. © 2000 Cancer Research Campaign


*British Journal of Cancer* (2000) **82**(1), 20–27
© 2000 Cancer Research Campaign
Article no. bjoc.1999.0871

### Phosphoglycerate mutase, 2,3-bisphosphoglycerate phosphatase, creatine kinase and enolase activity and isoenzymes in breast carcinoma

**N Durany[1], J Joseph[1], OM Jimenez[1], F Climent[1], PL Fernández[2], F Rivera[3] and J Carreras[1]**

[1]Unit of Biochemistry, IDIBAPS, Faculty of Medicine, University of Barcelona, Casanova 143, Barcelona 08036, Spain; [2]Pathological Anatomy Department and [3]Hormonal Laboratory, IDIBAPS, Hospital Clinı́c i Provincial, Villarroel 170, Barcelona 08036, Spain

**Summary** We have compared the levels of phosphoglycerate mutase (EC 5.4.2.1), 2,3-bisphosphoglycerate phosphatase (EC 3.1.3.13), creatine kinase (EC 2.7.3.2) and enolase (EC 4.2.1.11) activities and the distribution of their isoenzymes in normal breast tissue and in breast carcinoma. Tumour tissue had higher phosphoglycerate mutase and enolase activity than normal tissue. Creatine kinase activity was higher in seven out of 12 tumours. In contrast 2,3-bisphosphoglycerate phosphatase activity was lower. Phosphoglycerate mutase, enolase and 2,3-bisphosphoglycerate phosphatase presented greater changes in the oestrogen receptor-negative/progesterone receptor-negative breast carcinomas than in the steroid receptor-positive tumours. Determined by electrophoresis, type BB phosphoglycerate mutase, type BB creatine kinase and $\alpha\alpha$-enolase were the major isoenzymes detected in normal breast tissue. Types $\alpha\gamma$ and $\gamma\gamma$ enolase, types MB and MM phosphoglycerate mutase were detected in much lower proportions. In tumours a decrease of phosphoglycerate mutase isoenzymes possessing M-type subunit and some increase of enolase isoenzymes possessing $\gamma$-type subunit was observed. No detectable change was observed in the creatine kinase phenotype. © 2000 Cancer Research Campaign

**Key words:** 2,3-bisphosphoglycerate phosphatase; creatine kinase; enolase; phosphoglycerate mutase activity and isoenzymes; breast carcinoma

Phosphoglycerate mutase (PGM) (D-Phosphoglycerate 2,3-phosphomutase, EC 5.4.2.1), and enolase (2-phospho-D-glycerate hydrolyase, EC 4.2.1.11) are glycolytic enzymes that catalyse consecutive reversible reactions connecting the two ATP-generating reactions in the glycolytic pathway. PGM catalyses the conversion of 3-phosphoglycerate, product of the first ATP-generating reaction, into 2-phosphoglycerate, in the presence of the co-factor 2,3-bisphosphoglycerate. In addition to the main mutase activity, PGM possesses collateral 2,3-bisphosphoglycerate phosphatase (BPGP; EC 3.1.3.13) activity which is stimulated by 2-phosphoglycolate (reviewed in Fothergill-Gilmore and Watson, 1989). Enolase catalyses the conversion of 2-phosphoglycerate into phosphoenolpyruvate, substrate of the second ATP-generating glycolytic reaction (reviewed in Wold, 1971). Creatine kinase (CK) (ATP: creatine N-phosphotransferase, EC 2.7.3.2) is an ubiquitous enzyme that catalyses the reversible transphosphorylation reaction between ATP and creatine, generating ADP and phosphocreatine. It has a key role in the energy metabolism of cells with high and fluctuating energy demands, where it develops two main primary functions: to constitute a temporal energy buffer and a spatial energy buffer or energy transport system between intracellular sites of ATP production and sites of ATP consumption. In addition, CK develops other secondary functions: to prevent a rise in intracellular ADP avoiding an inactivation of ATPases, to avoid oscillations in the concentration of high-energy phosphates upon abrupt changes in workload, to provide appropriate local ATP/ADP ratios at subcellullar sites and to act as proton buffering system (reviewed in Walliman et al, 1992; Wyss et al, 1992).

All these enzymes possess tissue-specific isoenzymes. In mammalian tissues, there are three isoenzymes of PGM and three cytosolic isoenzymes of CK which result, in both cases, from the homodimeric and the heterodimeric combinations of two different subunits coded by separate genes and designated M (muscle) and B (brain). In early fetal life, type BB-PGM and type BB-CK are the only present forms. During myogenesis the isoenzyme phenotypes undergo transition, type BB-PGM and type BB-CK being replaced by the MM forms through the MB isoenzymes. In adult mammals, skeletal muscle contain almost exclusively type MM-PGM and type MM-CK, whereas type BB-PGM and type BB-CK are found in most other tissues. Only in heart are the three PGM and CK isoenzymes present in substantial amounts. In addition to the cytosolic CK subunits, mammalian tissues express two mitochondrial CK subunits (‘ubiquitous’ Mt-CK and ‘sarcomeric’ Mt-CK subunits) that form octameric and dimeric molecules (reviewed in Wallimann et al, 1992; Wyss et al, 1992; Carreras and Gallego, 1993; Durany and Carreras, 1996). In addition to PGM isoenzymes, in mammalian tissues there are other enzymes that have 2-phosphoglycolate-stimulated BPGP activity. One of them is the 2,3-bisphosphoglycerate synthase-phosphatase or 2,3-bisphosphoglycerate mutase (BPGM; EC 5.4.2.4), which is a homodimer of a subunit that possesses great homology with PGM subunits. Two other enzymes are heterodimers resulting from the combination of a BPGM subunit with a PGM subunit either type M or type B (reviewed in Carreras and Gallego, 1993). Enolase molecules are dimers composed of three distinct subunits coded by separate genes and designated $\alpha$ (liver), $\beta$ (muscle) and $\gamma$ (brain).

*Received 29 January 1999*
*Revised 26 May 1999*
*Accepted 7 July 1999*

*Correspondence to:* J Carreras

The αα isoenzyme exists in all fetal tissues and most adult mammalian tissues. ββ- and γβ-enolase are found predominantly in skeletal and heart muscle. γγ- and αγ-enolase are present mainly in nervous tissue and in tissues with neuroendocrine cells. They have been frequently designated as neuron-specific enolase (NSE) (Schmechel et al, 1978; Kato et al, 1983; Haimoto et al, 1985).

As a first step to investigate the alterations of the expression of the enzymes of energy metabolism in neoplastic tissues, in order to explain the underlying metabolic changes and to validate tumour marker enzymes and prognostic factors, we have studied the distribution of PGM, enolase, cytosolic CK and BPGP activities and isoenzymes in several human tumours. There is a wealth of information concerning the expression of CK and enolase activity and isoenzymes in neoplastic tissues. However, most data have been obtained by inmunohistochemical and inmunoassay techniques, and only a few reports have been published on the distribution of CK (reviewed in Foreback and Chu, 1981; Bais and Edwards, 1982; Griffiths, 1982; Nanji, 1983; Kanemitsu and Okigaki, 1984) and enolase isoenzyme proteins in tumours (reviewed in Taylor et al, 1983; Royds et al, 1985; Schmechel, 1985; Gerbitz et al, 1986; Marangos and Schmechel, 1987; Kaiser et al, 1989). Concerning the distribution of PGM isoenzymes in neoplastic tissues, only some data on brain tumours were available (Omenn and Cheung, 1974; Omenn and Hermodson, 1975). We have already published data concerning brain, lung, colon and liver tumours (Joseph et al, 1996, 1997; Durany et al, 1997*a*, 1997*b*). In the present study we present the results concerning normal breast and breast carcinomas, which largely confirm the findings from the other cancer types.

### MATERIALS AND METHODS

### Materials

Enzymes, substrates, co-factors and biochemicals were purchased from either Boehringer (Mannheim, Germany) or Sigma (St Louis, MO, USA). β-Mercaptoethanol was from Merck (Darmstadt, Germany) and bovine serum albumin was from Calbiochem (La Jolla, CA, USA). Other chemicals were reagent grade. Agar noble was obtained from Difco Laboratories (Detroit, MI, USA). Cellulose acetate strips were from Helena Laboratories (Beaumont, TX, USA) and agarose gels were from Ciba-Corning (Palo Alto, CA, USA).

### Tissue samples

Twenty-five breast carcinoma specimens were obtained from surgical resection. In 12 cases samples were available from non-neoplastic areas of the tissue. Samples were snap-frozen in liquid nitrogen and stored at –80°C. The specimens were provided by the Tissue Bank and by the Hormonal Laboratory of the Hospital Clínic i Provincial, with the approval of the Hospital Ethics Committee.

### Tissue extraction

Tissue extracts were prepared by homogenization in 3 vol (w/v) of cold 20 mM Tris–HCl buffer, pH 7.5, containing 1 mM EDTA and 1 mM β-mercaptoethanol with a Polytron homogenizer (Luzern, Switzerland) (position 5, 20 s). Cellular debris were removed by centrifugation at 4°C for 30 min at 12 500 ***g*** and the supernatants were used for the assay of enzyme activities and isoenzymes.

**Table 1** Levels of PGM activity in breast normal tissue and carcinomas

| | Normal tissue | | Tumour tissue | |
|---|---|---|---|---|
| **Case no.** | **U $g^{-1}$** | **U $mg^{-1}$** | **U $g^{-1}$** | **U $mg^{-1}$** |
| 1 | 2.4 | 0.18 | 3.9 | 0.24 |
| 2 | 1.8 | 0.15 | 6.0 | 0.46 |
| 3 | 2.1 | 0.19 | 8.9 | 0.36 |
| 4 | 5.7 | 0.30 | 15.9 | 0.80 |
| 5 | 1.8 | 0.08 | 16.8 | 0.65 |
| 6 | 0.5 | 0.03 | 4.2 | 0.33 |
| 7 | 0.6 | 0.05 | 6.8 | 0.50 |
| 8 | 0.8 | 0.04 | 9.6 | 0.48 |
| 9 | 1.0 | 0.05 | 9.6 | 0.38 |
| 10 | 0.5 | 0.03 | 3.9 | 0.20 |
| 11 | 0.5 | 0.05 | 3.4 | 0.23 |
| 12 | 0.4 | 0.04 | 3.1 | 0.23 |
| Mean ± s.e.m. | 1.5 ± 0.4 | 0.1 ± 0.02 | 7.6 ± 1.3 | 0.4 ± 0.05 |
| Median (range) | 0.9 (0.4–5.7) | 0.05 (0.03–0.3) | 6.4 (3.1–16.8) | 0.37 (0.2–0.8) |

The activity is expressed as units per g wet tissue and as units per mg of extracted protein. The comparisons are as follows: U $g^{-1}$: control vs carcinoma, $P < 0.0005$; U $mg^{-1}$: control vs carcinoma, $P < 0.0005$.

**Table 2** Levels of creatine kinase activity in breast normal tissue and carcinomas

| | Normal tissue | | Tumour tissue | |
|---|---|---|---|---|
| **Case no.** | **U $g^{-1}$** | **U $mg^{-1}$** | **U $g^{-1}$** | **U $mg^{-1}$** |
| 1 | 2.10 | 0.16 | 0.60 | 0.04 |
| 2 | 1.50 | 0.12 | 0.92 | 0.06 |
| 3 | 1.60 | 0.14 | 3.86 | 0.09 |
| 4 | 3.50 | 0.16 | 2.20 | 0.10 |
| 5 | 0.85 | 0.03 | 1.20 | 0.04 |
| 6 | 2.40 | 0.16 | 8.06 | 0.37 |
| 7 | 0.88 | 0.07 | 0.60 | 0.04 |
| 8 | 1.90 | 0.07 | 1.12 | 0.04 |
| 9 | 2.00 | 0.09 | 6.10 | 0.19 |
| 10 | 1.30 | 0.07 | 3.10 | 0.18 |
| 11 | 0.84 | 0.09 | 2.30 | 0.13 |
| 12 | 1.41 | 0.07 | 3.70 | 0.26 |
| Mean ± s.e.m. | 1.7 ± 0.2 | 0.1 ± 0.01 | 2.8 ± 0.67 | 0.12 ± 0.03 |
| Median (range) | 1.6 (0.84–3.5) | 0.09 (0.03–0.16) | 2.2 (0.6–8.06) | 0.09 (0.04–0.37) |

The activity is expressed as units per g wet tissue and as units per mg of extracted protein. The comparisons are as follows: U $g^{-1}$: control vs carcinoma, non-significant; U $mg^{-1}$: control vs carcinoma, non-significant.

### Enzyme and protein assays

PGM, BPGP, CK and enolase activities were determined as previously described (Durany and Carreras, 1996; Joseph et al, 1996, 1997). Enzyme activities were expressed as U $g^{-1}$ wet tissue and as U $mg^{-1}$ protein (1 Unit = 1 μmol substrate converted per min). Protein was determined by the method of Bradford (1976), using bovine serum albumin as a standard.

### Isoenzyme analysis

The methods previously described were used to evaluate PGM isoenzymes by cellulose acetate electrophoresis (Durany and Carreras, 1996) and CK and enolase isoenzymes by agarose gel electrophoresis (Joseph et al, 1996, 1997).

© 2000 Cancer Research Campaign 

**Table 3** Levels of enolase activity in breast normal tissue and carcinomas

| | Normal tissue | | Tumour tissue | |
|---|---|---|---|---|
| **Case no.** | **U g$^{-1}$** | **U mg$^{-1}$** | **U g$^{-1}$** | **U mg$^{-1}$** |
| 1 | 0.95 | 0.07 | 1.6 | 0.10 |
| 2 | 1.10 | 0.09 | 4.5 | 0.29 |
| 3 | 1.10 | 0.10 | 10.0 | 0.26 |
| 4 | 8.30 | 0.38 | 7.4 | 0.36 |
| 5 | 0.90 | 0.04 | 3.3 | 0.10 |
| 6 | 1.03 | 0.07 | 4.4 | 0.20 |
| 7 | 0.62 | 0.05 | 3.7 | 0.27 |
| 8 | 1.99 | 0.07 | 2.7 | 0.10 |
| 9 | 3.30 | 0.16 | 14.8 | 0.47 |
| 10 | 1.25 | 0.07 | 4.7 | 0.27 |
| 11 | 0.71 | 0.07 | 4.6 | 0.25 |
| 12 | 1.26 | 0.06 | 4.4 | 0.32 |
| Mean ± s.e.m. | 1.9 ± 0.6 | 0.1 ± 0.026 | 5.51 ± 1.05 | 0.25 ± 0.03 |
| Median (range) | 1.1 (0.6–8.3) | 0.07 (0.04–0.38) | 4.5 (1.6–14.8) | 0.26 (0.1–0.47) |

The activity is expressed as units per g wet tissue and as units per mg of extracted protein. The comparisons are as follows: U g$^{-1}$: control vs carcinoma, $P < 0.002$; U mg$^{-1}$: control vs carcinoma, $P < 0.001$.

**Table 4** Levels of BPGP activity in breast normal tissue and carcinomas

| | Normal tissue | | Tumour tissue | |
|---|---|---|---|---|
| **Case no.** | **mU g$^{-1}$** | **mU mg$^{-1}$** | **mU g$^{-1}$** | **mU mg$^{-1}$** |
| 1 | 60.5 | 4.5 | 50.1 | 3.2 |
| 2 | 57.0 | 4.2 | 39.9 | 3.1 |
| 3 | 63.0 | 5.7 | 54.0 | 2.2 |
| 4 | 83.2 | 4.4 | 55.1 | 2.8 |
| 5 | 79.9 | 3.6 | 60.1 | 2.4 |
| 6 | 64.8 | 4.3 | 39.9 | 3.1 |
| 7 | 14.9 | 1.3 | 10.2 | 0.8 |
| 8 | 117.0 | 5.5 | 27.3 | 1.3 |
| 9 | 93.0 | 5.0 | 64.8 | 2.6 |
| 10 | 32.4 | 2.2 | 24.9 | 1.5 |
| 11 | 74.7 | 8.0 | 71.4 | 4.8 |
| 12 | 19.8 | 2.1 | 12.3 | 1.2 |
| Mean ± s.e.m. | 63 ± 8.6 | 4.2 ± 0.5 | 42.5 ± 5.8 | 2.4 ± 0.3 |
| Median (range) | 63 (15–117) | 4.3 (1.3–8.0) | 45 (10–71) | 2.5 (0.8–4.8) |

The activity is expressed as units per g wet tissue and as units per mg of extracted protein. The comparisons are as follows: U g$^{-1}$: control vs carcinoma, $P < 0.0005$; U mg$^{-1}$: control vs carcinoma, $P < 0.0015$.

### Inhibition of M-CK subunit

Inhibition of M-CK subunit by M-CK antibodies was performed as previously described (Joseph et al, 1997).

### Oestrogen and progesterone receptor analysis

Oestrogen receptor (ER) and progesterone receptor (PR) were immunohistochemically analysed by the stretavidin–biotin–alkaline phosphatase method as previously reported (Jares et al, 1997). Cases were evaluated with a modified HSCORE system (McCarty et al, 1985) by multiplying the stratified percentage of positive cells (1–33% = 1; 34–66% = 2; 67–100% = 3) by the average intensity of each case (1 to 3). A case was considered R-positive when the score was 1 or higher. Normal glands and ducts were used as internal positive controls. As a negative control, primary antibodies were replaced by unrelated monoclonal antibodies and by phosphate-buffered saline (PBS).

### Statistical analysis

For statistical evaluation, the Wilcoxon *T*-test was used to compare enzyme activities in tumour and control tissue. The Kruskal–Wallis test (non-parametric analysis of variance) was employed to compare enzyme activity levels among different tumour groups and control. The differences between groups were located using the Mann–Whitney *U*-test. All *P*-values are two-tailed. Values are reported as mean ± s.e.m. and as median and range. Data were analysed by Instat statistical software.

### RESULTS AND DISCUSSION

### Distribution of PGM, CK, enolase and BPGP activities

Tables 1–4 summarize the levels of total PGM, CK, enolase and BPGP activities in breast normal tissue and tumours. Figures 1–3 present some of the isoenzyme patterns determined by electrophoresis, and Tables 5 and 6 summarize the distribution of the isoenzymes in normal and tumour tissues. Opinions differ widely as to how is the most useful way to express the enzyme activity in tissue when diverse tissues are being compared (Crabtree et al, 1979). Changes in tissue structure, in the proportion of parenchymal cells and in the active metabolic mass of tissue in tumours could mask the intracellular levels of enzyme activity when the activity is expressed as a function of the wet tissue mass. Changes in the total amount of extractable protein resulting from the neoplastic transformation could mask the levels of enzyme activity when it is expressed as a function of the extracted protein. Therefore we have determined the enzyme activities as a function of both wet tissue mass and extracted protein.

As shown, normal breast tissue possesses similar levels of PGM, CK and enolase activities, and much lower levels of 2-phosphoglycolate-stimulated BPGP activity, which is in agreement with the different functions of these enzymes. Whereas PGM and enolase are enzymes of the main glycolytic pathway and CK is directly involved in energy metabolism, BPGP participates in the 2,3-bisphosphoglycerate bypass (or Rapoport-Luebering shunt), a collateral deviation of glycolysis (Rapoport, 1968).

Breast tumours have significantly higher PGM (Table 1) and enolase (Table 3) activity levels than the corresponding normal tissues, which agree with data reported on other glycolytic enzymes. It was found that enolase, hexokinase, phosphofructokinase, pyruvate kinase and aldolase activities were elevated in breast carcinoma in comparison to benign breast diseases and normal breast tissue, although great intratumoural and intertumoural heterogeneity was observed (Hennipman et al, 1987, 1988).

It has been reported (Meyer et al, 1980; Tsung, 1983; Lakatua et al, 1986) that the levels of CK activity in breast carcinoma are higher than in normal breast tissue. As shown in Table 2, we have found higher CK activity in only seven out of 12 cases. In one of these cases (no. 3), CK activity in neoplastic tissue was lower than in normal tissue when the activity was reported as a function of the extracted protein. However, in this case the extracted protein from the tumour tissue was abnormally high (3.6-fold that of the normal tissue). In all other cases, although the extracted protein from tumoural tissue was higher than the extracted protein from normal

© 2000 Cancer Research Campaign

**Table 5** Distribution of PGM isoenzymes in breast normal tissue and carcinomas

| Case no. | Normal tissue MM | Normal tissue MB | Normal tissue BB | Tumour tissue MM | Tumour tissue MB | Tumour tissue BB |
|---|---|---|---|---|---|---|
| 1 | 1 | 11 | 88 | 0 | 0 | 100 |
| 2 | 0 | 9 | 91 | 0 | 0 | 100 |
| 3 | 0 | 0 | 100 | 0 | 2 | 98 |
| 4 | 0 | 4 | 96 | 0 | 0 | 100 |
| 5 | 0 | 0 | 100 | 0 | 0 | 100 |
| 6 | 0 | 2 | 98 | 0 | 0 | 100 |
| 7 | 0 | 4 | 96 | 0 | 1 | 99 |
| 8 | 0 | 2 | 98 | 0 | 0 | 100 |
| 9 | 0 | 4 | 96 | 0 | 0 | 100 |
| 10 | 0 | 3 | 97 | 0 | 0 | 100 |
| 11 | 0 | 9 | 91 | 0 | 0 | 100 |
| 12 | 0 | 0 | 100 | 0 | 0 | 100 |
| Mean ± s.e.m. | 0.08 ± 0.08 | 4 ± 1.0 | 95.9 ± 1.1 | 0 ± 0 | 0.25 ± 0.1 | 99.7 ± 0.1 |
| Median (range) | 0 (0–1) | 3.5 (0–11) | 96.5 (88–100) | 0 (0–0) | 0 (0–2) | 100 (98–100) |

The results are expressed as percentage of the total PGM activity on electrophoresis. No statistical significant differences were observed.

**Table 6** Distribution of enolase isoenzymes in breast normal tissue and carcinomas

| Case no. | Normal tissue αα | Normal tissue αγ | Normal tissue γγ | Tumour tissue αα | Tumour tissue αγ | Tumour tissue γγ |
|---|---|---|---|---|---|---|
| 1 | 86 | 12 | 2 | 76 | 20 | 1 |
| 2 | 89 | 9 | 2 | 82 | 15 | 3 |
| 3 | 90 | 9 | 1 | 67 | 29 | 4 |
| 4 | 83 | 16 | 1 | 81 | 18 | 1 |
| 5 | 88 | 12 | 0 | 90 | 10 | 0 |
| 6 | 85 | 13 | 2 | 54 | 35 | 11 |
| 7 | 97 | 2 | 1 | 85 | 13 | 2 |
| 8 | 83 | 14 | 3 | 79 | 18 | 3 |
| 9 | 85 | 13 | 2 | 81 | 16 | 3 |
| 10 | 89 | 10 | 1 | 84 | 15 | 1 |
| 11 | 90 | 10 | 0 | 88 | 11 | 1 |
| 12 | 86 | 13 | 1 | 80 | 18 | 2 |
| Mean ± s.e.m. | 87.5 ± 1.1 | 11.1 ± 1.0 | 1.3 ± 0.25 | 78.9 ± 2.8 | 18.1 ± 2.8 | 2.6 ± 0.8 |
| Median (range) | 87.0 (83–97) | 12.0 (2–16) | 1.0 (0–3) | 81.0 (54–90) | 17.0 (10–35) | 2.0 (0–11) |

The results are expressed as percentage of the total enolase activity on electrophoresis. The comparisons are as follows: αα-enolase: control vs carcinoma, $P < 0.0015$; αγ-enolase: control vs carcinoma, $P < 0.0015$; γγ-enolase: control vs carcinoma, $P < 0.05$.

tissue, it did not surmount a 1.5-fold increase.

In contrast to PGM, enolase and CK, the levels of 2-phosphoglycolate-stimulated BPGP activity in breast tumours are lower than in normal breast tissue (Table 4). The fact that breast carcinomas present opposite changes in the levels of PGM activity and of 2-phosphoglycolate-stimulated BPGP activity indicates that in tumoural tissue change both the concentration of PGM and the concentration of the other enzymes that also have BPGP activity (BPGM and BPGM-PGM hybrid). Similar results have been found in lung, colon and liver carcinomas (Durany et al, 1997), which indicates that in all these tumours could decrease the metabolic flux through the 2,3-bisphosphoglycerate shunt, and change the intracellular concentration of 2,3-bisphosphoglycerate. It is known that this metabolite, in addition to act as cofactor of PGM, ‘in vitro’ inhibits several enzymes involved in the carbohydrate and adenine nucleotide metabolism, although the physiological significance of these inhibitory effects is uncertain (reviewed in Carreras et al, 1986).

### Distribution of PGM, CK and enolase isoenzymes

No previous data exist on the distribution of PGM isoenzymes in breast normal tissue and tumours. Our results (Table 5 and Figure 1) show that in normal breast tissue type BB-PGM is the major PGM isoenzyme. Type MB-PGM is present in very small proportion (mean value: 4% of the total PGM activity) and type MM-PGM is generally not detected. In breast carcinomas a decrease of PGM isoenzymes possessing M-type subunit is observed: in most speciments of breast tumours only type BB-PGM is detected. As indicated in the Introduction, in mammals, type B subunit is the only PGM subunit expressed in early fetal life. During development, in muscle, brain and sperm cells a switch from type B to type M PGM subunit occurs, but only in skeletal muscle and sperm cells a complete transition takes place. As a consequence, in adult mammals, skeletal muscle and sperm cells contain almost exclusively type MM-PGM, whereas in heart and in some brain areas, types MB and BB-PGM are present in substantial amounts.

© 2000 Cancer Research Campaign

**Table 7** CK, PGM, enolase and BPGP activities as a function of receptor status

| Activity | Tissue | Receptor status | No. | U g$^{-1}$ tissue | | U mg$^{-1}$ protein | |
|---|---|---|---|---|---|---|---|
| | | | | Mean ± s.e.m. | Median (range) | Mean ± s.e.m. | Median (range) |
| CK | Normal | | 12 | 1.70 ± 0.22 | 1.50 (0.84–3.5) | 0.10 ± 0.013 | 0.09 (0.03–0.16) |
| | Tumoural | ER+ PR+ | 12 | 2.32 ± 0.47 | 2.15 (0.6–6.7) | 0.12 ± 0.026 | 0.085 (0.04–0.34) |
| | Tumoural | ER– PR– | 10 | 1.93 ± 0.52 | 1.30 (0.6–6.1) | 0.08 ± 0.018 | 0.064 (0.04–0.19) |
| PGM | Normal | | 12 | 1.50 ± 0.43 | 0.90 (0.4–5.7) | 0.10 ± 0.025 | 0.05 (0.03–0.30) |
| | Tumoural | ER+ PR+ | 10 | 5.44 ± 1.02 | 3.85 (2.6–11.9) | 0.26 ± 0.038 | 0.23 (0.10–0.46) |
| | Tumoural | ER– PR– | 13 | 9.36 ± 1.18 | 9.60 (3.4–16.8) | 0.43 ± 0.050 | 0.44 (0.18–0.80) |
| Enolase | Normal | | 12 | 1.87 ± 0.62 | 1.10 (0.62–8.3) | 0.10 ± 0.027 | 0.07 (0.04–0.38) |
| | Tumoural | ER+ PR+ | 10 | 4.20 ± 0.60 | 4.45 (1.6–8.1) | 0.20 ± 0.027 | 0.20 (0.09–0.32) |
| | Tumoural | ER– PR– | 10 | 7.38 ± 1.36 | 6.33 (2.7–14.8) | 0.32 ± 0.043 | 0.34 (0.10–0.47) |
| BPGP | Normal | | 12 | 63.3 ± 8.61 | 64.0 (14.9–117) | 4.23 ± 0.52 | 4.35 (1.30–8.0) |
| | Tumoural | ER+ PR+ | 7 | 60.5 ± 11.08 | 63.0 (12.3–97) | 3.24 ± 0.46 | 3.20 (1.20–4.8) |
| | Tumoural | ER– PR– | 7 | 41.7 ± 7.86 | 50.0 (10.2–64.8) | 2.00 ± 0.297 | 2.40 (0.80–2.80) |

The comparisons are as follows. CK, U g$^{-1}$: ER+PR+ vs normal, ER–PR– vs normal, and ER+PR+ vs ER–PR–, not significant. U mg$^{-1}$: ER+PR+ vs normal, ER–PR– vs normal, and ER+PR+ vs ER–PR–, not significant. PGM, U g$^{-1}$: ER+PR+ vs normal, $P < 0.0001$; ER–PR– vs normal, $P < 0.0001$; ER+PR+ vs ER–PR–, $P < 0.05$. U mg$^{-1}$: ER+PR+ vs normal, $P < 0.05$; ER–PR– vs normal, $P < 0.0001$; ER+PR+ vs ER–PR–, $P < 0.05$. Enolase, U g$^{-1}$: ER+PR+ vs normal, $P < 0.05$; ER–PR– vs normal, $P < 0.05$; ER+PR+ vs ER– PR–, not significant. U mg$^{-1}$: ER+PR+ vs normal, $P < 0.05$; ER–PR– vs normal, $P < 0.05$; ER+ PR+ vs ER–PR–, $P < 0.05$. BPGP, mU g$^{-1}$: ER+PR+ vs normal, ER–PR– vs normal, and ER+PR+ vs ER–PR– not significant. mU mg$^{-1}$: ER–PR– vs normal, $P < 0.05$; ER+PR+ vs normal and ER+PR+ vs ER–PR–, not significant.

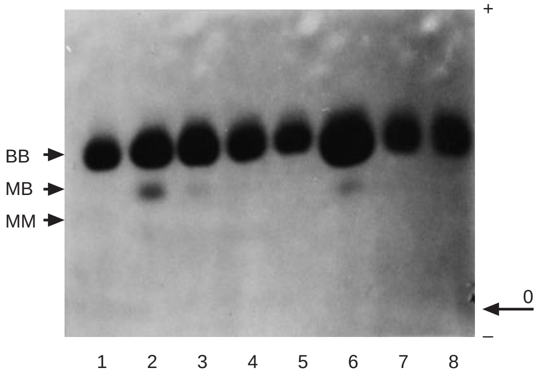

**Figure 1** Electrophoretograms of PGM isoenzymes in extracts of human breast normal tissue and carcinomas. Lanes 1 and 2, case no. 1; lanes 3 and 4, case no. 3; lanes 5 and 6, case no. 4; lanes 7 and 8, case no. 5. Uneven lanes, tumour tissue; even lanes, normal tissue. Experimental conditions were those described in Material and Methods

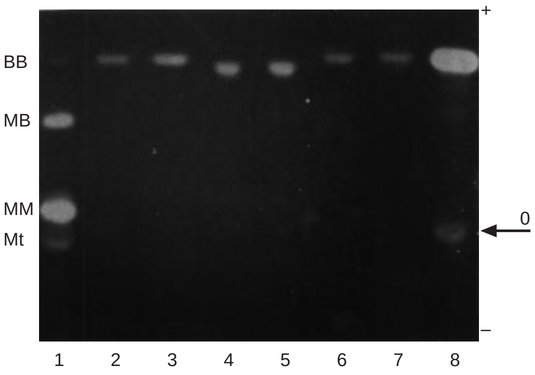

**Figure 2** Electrophoretograms of CK isoenzymes in extracts of human breast normal tissue and carcinomas. Lane 1, heart; lane 8, brain; lanes 2 and 3, case no. 3; lanes 4 and 5, case no. 4; lanes 6 and 7, case no. 5. Even lanes, tumour tissue; uneven lanes, normal tissue. Experimental conditions were those described in Material and Methods

The expression of type M-PGM subunit is almost null in the other adult mammalian tissues, although in some of them the hybrid type MB-PGM is detected in small proportion (Durany and Carreras, 1996; Durany et al, 1997*a*, 1997*b*). Therefore, the decrease in the expression of type M-PGM subunit in breast neoplastic cells now reported can be interpreted as a return to a more undifferentiated isoenzyme pattern. This fact probably does not have any physiological meaning, since the proportion of PGM isoenzymes containing type M subunit in normal breast tissue is very low and all PGM isoenzymes possess similar kinetic properties (Bartrons and Carreras, 1982).

By immunoperoxidase staining (Wold et al, 1981) only type B-CK subunit has been detected in normal breast ductal epithelium, and by electrophoresis (Tsung, 1983) and ion-exchange chromatography (Lakatua et al, 1986) it has been found that BB-CK was the predominant CK isoenzyme present in normal breast tissue, although MB- and MM-CK isoenzymes were found, at much lower proportion, in some specimens. The CK isoenzyme pattern of breast carcinomas, determined by electrophoresis (Tsung, 1983), by ion-exchange chromatography (Kaye et al, 1981; 1986; Tsung, 1983; Lakatua et al, 1986; Scambia et al, 1986*b*) and by immunoreactivity (Zarghami et al, 1995) has been found to vary greatly (from 100% BB-CK to 100% MM-CK), although most tumours showed preponderance of the BB-CK form.

As shown in Figure 2, we have detected only type BB-CK in most specimens of both normal and tumour breast tissue. Neither MM-CK nor MB-CK were detected, but it has to be noted that very low levels of CK (less than 5% of the total CK activity) would not be detected in our electrophoretic analysis. In some cases, in overloaded and overstained electrophoretograms, it was found a cathodic band migrated as Mt-CK and was not affected by incubation with anti M-CK antibodies (not shown). Mt-CK is known to strongly bind to the outer face of the inner mitochondrial membrane. To be quantitatively detached requires phosphate buffer, high molarity and high pH (Wiss et al, 1982). Our extraction procedure (low buffer strength, no salt, neutral pH) was

 *© 2000 Cancer Research Campaign*

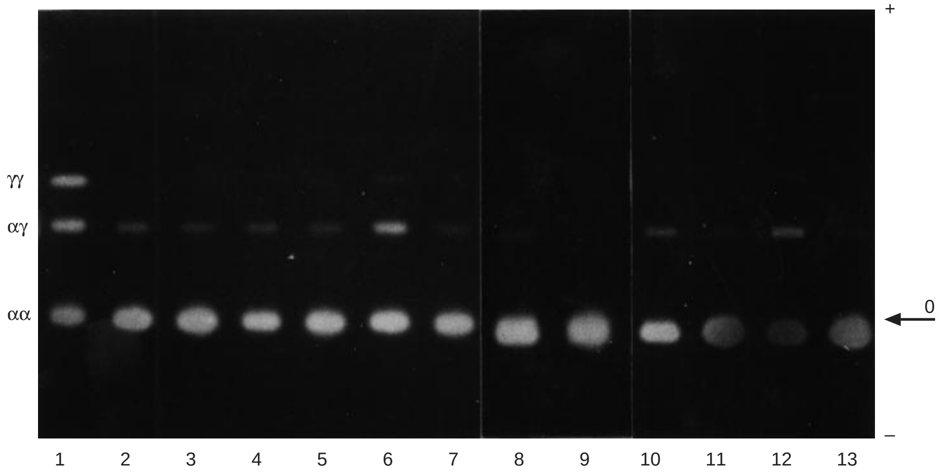

**Figure 3** Electrophoretograms of enolase isoenzymes in extracts of human breast normal tissue and carcinomas. Lane 1, brain; lanes 2 and 3, case no. 1; lanes 4 and 5, case no. 2; lanes 6 and 7, case no. 3; lanes 8 and 9, case no. 9, lanes 10 and 11, case no. 12; lanes 12 and 13, case no. 6. Even lines, tumour tissue; uneven lines, normal tissue. Experimental conditions were those described in Material and Methods selected to extract only the CK cytosolic isoenzymes but, as we have previously verified (Durany et al, 1997*b*; Joseph et al, 1997), it extracts also some Mt-CK.

Some of the conflicting results on the distribution of CK isoenzymes in breast could be explained by the confusion created by adenylate kinase, Mt-CK or BB-CK. Adenylate kinase and Mt-CK can contaminate the MM-CK electrophoretic band and by ion-exchange chromatography co-elute with MM-CK (Klein and Jeunelot, 1978; Lindsey et al, 1978; Desjardins, 1982; Urdal et al, 1983). From BB-CK may appear an artefactual band with an electrophoretic mobility similar to that of MB-CK (Chastian et al, 1988). Moreover, by electrophoresis (Meyer et al, 1980) in breast tumours a variant of CK-isoenzymes has been found which migrated anodal to MM-CK. This variant, that was present only in small quantities in normal breast tissue, was unaffected by antibodies specific for both the M- and B-CK subunits (Meyer et al, 1980).

As shown in Figure 3 and summarized in Table 6, in normal breast tissue αα-enolase is the predominant enolase isoenzyme. αγ-enolase is present in much lower proportion (2–16%) and the levels of γγ-enolase are very low (0–3%). Breast tumours possess a distribution of enolase isoenzymes similar to that of normal tissue, although the proportion of the isoenzymes possessing type γ subunit is higher. αγ- and γγ-enolase represent 8–35% and 0–11% of the total enolase activity respectively. No additional correlation is observed between activity changes and changes of the isoenzyme pattern when single cases are analysed. These results agree with data published by others. By cellulose acetate electrophoresis, αα-enolase was found to be the major enolase isoenzyme in normal breast tissue and in breast carcinomas (Hennipman et al, 1987). By ion-exchange chromatography NSE was found to represent 4–10% of the total enolase activity in four breast carcinomas (Pahlman et al, 1986). By immunohistochemistry no positive staining for γ-enolase was found in normal breast tissue (Vinores et al, 1984; Nesland et al, 1986*b*, 1987, 1988), but a high proportion of γ-enolase was detected in breast carcinoma (Vinores et al, 1984; Haimoto et al, 1985; Nesland et al, 1985, 1986*a*, 1986*b*, 1988, 1991*a*, 1991*b*; Wilander et al, 1987; Erikstein et al, 1988; Ingelman-Sundberg et al, 1989; Lilleng et al, 1992; Scopsi et al, 1992; Matsushima et al, 1994). By radioimmunoassay γ-enolase was also detected in breast carcinoma cell lines (Reeve et al, 1986; Zeltzer et al, 1986). We have previously found in carcinoid tumours of the lung a change of enolase phenotype similar to that now reported in breast carcinoma, in contrast to colon, liver and non-endocrine lung tumours, in which a decrease of the expression of γ-type enolase subunit was observed (Durany et al, 1997*a*).

### Correlation of the enzyme alterations and the steroid receptor status

BB-CK, which is a major oestrogen-regulated enzyme, has been found to be oestrogen-responsive in experimental mammary tumours (Kaye et al, 1981, 1986) as well as in human breast cancer (Kaye, 1983; Scambia et al, 1986*a*, 1986*b*). However, the literature data on the correlation between the levels of CK and the ER status of the breast neoplastic tissue is controversial. Two research groups (Kaye et al, 1981, 1986; Lakatua and Mohammed, 1986) reported lack of correlation between total CK activity or BB-CK activity and the concentration of either ER or PR in human breast tumour samples. Scambia et al (1986*b*) found total CK activity to be significantly higher in ER-positive/PR-positive and ER-negative/PR-positive tumours, although no correlation was found between ER or PR content and total CK or BB-CK activity. The same group in a second report (Scambia et al, 1988) showed a positive relationship between BB-CK positivity and ER content in ER-rich breast tumours. Winstend and Hopps (1995) reported correlation between high levels of total CK activity and positive ER levels. Zarghami et al (1995, 1996) reported association between BB-CK and ER but not PR, although in only one of the reports (Zarghami et al, 1995) did this association reach statistical significance.

As shown in Table 7, our results indicate no correlation between total CK activity and the ER-positive or PR-positive status of the breast tumours. Discrepancies in the reported data on correlation between enzymatic activity and steroid receptor status of breast carcinomas do not only exist in the case of CK (Messeri et al, 1983, and references therein). They could derive from the different number of patients studied and from the different methods used to assay the receptors (Zarghami et al, 1996) and the enzyme activity

© 2000 Cancer Research Campaign 

(Messeri et al, 1983). An additional factor of discrepancy could be the great intratumoural heterogeneity existent in breast neoplastic tissue. Immunohistochemical localization of BB-CK have demonstrated the frequent presence of positive and negative cells in close proximity (Scambia et al, 1988). Biochemical determination of the activities and of the isoenzyme patterns of several glycolytic enzymes have shown regional heterogeneity (Hennipman et al, 1988). Heterogeneity has been reported also in receptor status of breast tumours (Klinga et al, 1982; Osborne, 1985; Pertschuk et al, 1985).

PGM has not been proved to be regulated by steroid hormones. However, as summarized in Table 7, a correlation appears to exist between the total PGM activity and the steroid receptor status of the breast carcinomas. The ER-negative/PR-negative carcinomas possess significantly higher PGM activity than the ER-positive/PR-positive tumours.

The mean value of total enolase activity of ER-negative/PR-negative breast carcinomas is higher than that of ER-positive/PR-positive breast tumours, although the difference between both groups of tumours only reach statistical significance when the activity is expressed a function of the extracted protein (Table 7). It is known that the majority of tumours possessing NSE have immunoreactivity for several hormone receptors. Among the breast tumours, it was found that NSE-positive carcinomas were more frequently ER-positive than the NSE-negative ones, and that the ER levels were higher in the NSE-positive samples (Nesland et al, 1985; Erikstein et al, 1988). Although the number of tumours studied by us is small, our results agree with this finding. As shown in Table 6, four out of the five ER-positive/PR-positive tumours (cases 1, 2, 3, 11 and 12) are included in the group of tumours with higher proportion of NSE.

As indicated above (Table 4), in contrast to the PGM and the enolase activity, the 2-phosphoglycolate-stimulated BPGP activity in breast carcinomas is lower than in the corresponding normal breast tissue. When the steroid receptor status of the tumour samples is considered (Table 7), it is evident than only the ER-negative/PR-negative group of carcinomas possesses statistically significant lower BPGP activity than the group of normal tissues.

### CONCLUSION

It is concluded that PGM, enolase, CK and BPGP activities are differently affected in breast carcinomas. Whereas the PGM, enolase and CK activities increase in tumours, the BPGP activity decreases. A correlation appears to exist between the steroid receptor status of the carcinomas and the changes of PGM, enolase and BPGP activities. These three enzymatic activities present greater changes in the ER-negative/PR-negative carcinomas than in the ER-positive/PR-positive tumours. No correlation has been found between the receptor status of the carcinomas and the levels of CK activity.

Tumoural breast tissue shows minor changes in the PGM and enolase isozyme patterns. In the PGM phenotype we have detected a decrease in the expression of M-type subunit, which is typical of differentiated muscle and sperm cells, and which is present in very low levels in normal breast tissue. In the enolase phenotype we have observed some increase of the isoenzymes possessing γ-type subunit, which is mainly expressed in nervous tissue and in neuroendocrine cells. No change has been observed in CK phenotype. None of the changes detected in breast tumours is great enough to be used as good breast tumour marker.

### ACKNOWLEDGEMENTS

This work was supported by FIS (Grant 93/0573), by the Generalitat de Catalunya (Grant GRQ 94-1036) and the Marató TV3 (Grant 38/95s2).

### REFERENCES

Bais R and Edwards J (1982) Creatine kinase. *Crit Rev Clin Lab Sci* **16**: 291–335

Bartrons R and Carreras J (1982) Purification and characterization of phosphoglycerate mutase isoenzymes from pig heart. *Biochim Biophys Acta* **708**: 167–177

Bradford M (1976) A rapid and sensitive method for the quantification of microgram quantities of protein utilizing the principle of protein-dye binding. *Anal Biochem* **72**: 248–254

Carreras J and Gallego C (1993) Metabolism of 2,3-bisphosphoglyceric acid in erythroid cells and tissues of vertebrates. *Trends Comp Biochem Physiol* **1**: 421–450

Carreras J, Bartrons R, Climent F and Cussó R (1986) Bisphosphorylated metabolites of glycerate, glucose and fructose: functions, metabolism and molecular patholgy. *Clin Biochem* **19**: 348–358

Chastain S, Ketchum C and Grizzle W (1988) Stability and electrophoretic characteristics of creatine kinase BB extracted from human brain and intestine. *Clin Chem* **334**: 489–492

Crabtree B, Leech A and Newsholme A (1979) Measurement of enzyme activities in crude extracts of tissues. In: *Techniques in the Live Sciences*, Vol B2/1, Kornberg HL, Metcalfe JC, Northcote DH, Pogson CI and Tipton KF (eds), pp. B211/1–B211/37. Elservier/North Holland Biomedical Press: Amsterdam

Desjardins P (1982) Characterization of an atypical creatine kinase from human heart tissue, with properties similar to those of mitochondrial creatine kinase. *Clin Chim Acta* **121**: 67–78

Durany N and Carreras J (1996) Distribution of phosphoglycerate mutase isozymes in rat, rabbit and human tissues. *Comp Biochem Physiol* **113**: 217–223

Durany N, Joseph J, Campo E, Molina R and Carreras J (1997*a*) Phosphoglycerate mutase, 2,3-bisphosphoglycerate phosphatase and enolase activity and isoenzymes in lung, colon and liver carcinomas. *Br J Cancer* **76**: 969–977

Durany N, Joseph J, Cruz-Sanchez F and Carreras J (1997*b*) Phosphoglycerate mutase, 2,3-bisphosphoglycerate phosphatase and creatine kinase activity and isoenzymes in human brain tumours. *Br J Cancer* **76**: 1139–1149

Erikstein B, Nesland J, Ottestad L, Lund E, Johannessen J (1988) Neuron-specific enolase-positive breast carcinomas. *Histol Histopathol* **3**: 97–102

Foreback C and Chu J (1981) Creatine kinase isoenzymes: electrophoretic and quantitative measurements. *Crit Rev Clin Lab Sci* **15**: 187–230

Fothergill-Gilmore L and Watson H (1989) The phosphoglycerate mutases. *Adv Enzymol* **62**: 227–313

Gerbitz K, Summer J and Schumacher I (1986) Enolase isoenzymes as tumour markers. *J Clin Chem Clin Biochem* **24**: 1009–1016

Griffiths J (1982) Creatine kinase isoenzyme 1. *Clin Lab Med* **2**: 493–506

Haimoto J, Takahashi Y, Koshikawa T, Nagura H and Kato K (1985) Immunohistochemical localization of γ-enolase in normal human tissues other than nervous and neuroendocrine tissues. *Lab Invest* **52**: 257–263

Hennipman A, Smits J, Van Oirschot B, Van Houwelingen J, Rijksen G, Neyt J, Van Unnik J and Staal G (1987) Glycolitic enzymes in breast cancer, benign breast disease and normal breast tissue. *Tumor Biol* **8**: 251–263

Hennipman A, Van Oirschot B, Smits J, Rijksen G and Staal G (1988) Heterogeneity of glycolytic enzyme activity and isozyme composition of pyruvate kinase in breast cancer. *Tumor Biol* **9**: 178–189

Ingelman-Sundberg H, Wikström B, Stormby N, Sundelin P and Hjerpe A (1989) Immunocytochemical reactivity of breast cancer tissue with antibodies to neuron-specific enolase and an adenocarcinoma-associated glycolipid antigen. *Virchows Archiv A Pathol Anat* **415**: 539–544

Jares P, Rey M, Fernández P, Campo E, Nadal A, Muñoz M, Mallofré C, Muntané J, Nayach I, Estapé J and Cardesa A (1997) Cyclin D1 and retinoblastoma gene expression in human breast carcinoma: correlation with tumor proliferation and estrogen receptor status. *J Pathol* **182**: 160–166

Joseph J, Cardesa A and Carreras J (1997) Creatine kinase activity and isoenzymes in lung, colon and liver carcinomas. *Br J Cancer* **76**: 100–105

Joseph J, Cruz-Sánchez F and Carreras J (1996) Enolase activity and isoenzyme distribution in human brain regions and tumors. *J Neurochem* **66**: 2484–2490

Kaiser E, Kuzmits R, Pregant P, Burghuber O and Worofka W (1989) Clinical biochemistry of neuron specific enolase. *Clin Chim Acta* **183**: 13–32

© 2000 Cancer Research Campaign

Kanemitsu F and Okigaki T (1988) Creatine kinase: a review. *J Cromatog* **429**: 399–417

Kato K, Ishiguro Y and Ariyoshi Y (1983) Enolase isozymes as disease markers: distribution of three enolase subunits ($\alpha$, $\beta$ and $\gamma$) in various human tissues. *Disease Markers* **1**: 213–220

Kaye A (1983) Enzyme induction by strogen. *J Steroid Biochem* **19**: 33–40

Kaye A, Reiss N, Shaer A, Sluyser M, Iacobelli S, Amroch D and Soffer Y (1981) Estrogen responsive creatine kinase in normal and neoplastic cells. *J Steroid Biochem* **15**: 69–75

Kaye A, Hallowes R, Cox S and Sluyser M (1986) Hormone-responsive creatine kinase in normal and neoplastic mammary glands. *Ann NY Acad Sci* **464**: 218–230

Klein B and Jeunelot C (1978) Anion-exchange chromatography of erythrocytic and muscle adenylate kinase and its effect on the serum creatine kinase isoenzyme assays. *Clin Chem* **24**: 2168–2170

Klinga K, Kaufman M, Runnebaum B and Kubli F (1982) Distribution of estrogen and progesterone receptors in primary tumor and lymphnodes in individual patients with breast cancer. *Oncology* **39**: 337–339

Lakatua D and Mohammed R (1986) Estrogen and progesterone receptors and creatine kinase isoenzymes in human breast cancer. *Clin Chem* **32**: 2103–2104

Lilleng R, Hagmar B and Nesland J (1992) C-*erb*B-2 protein and neuroendocrine expression in intraductal carcinomas of the breast. *Mod Pathol* **5**: 41–47

Lindsey G and Diamond E (1978) Evidence for significant quantities of creatine kinase MM isoenzyme in human brain. *Biochim Biophys Acta* **524**: 78–84

McCarty K Jr, Miller L, Cox E, Konrath J and McCarty K (1985) Estrogen receptor analyses. Correlation of biochemical and immunohistochemical methods using monoclonal antireceptor antibodies. *Arch Pathol Lab Med* **109**: 716–722

Marangos P and Schmechel D (1987) Neuron specific enolase, a clinically useful marker for neurons and nonendocrine cells. *Annu Rev Neurosci* **10**: 269–295

Matsushima S, Mori M, Adachi Y, Matsukuma A and Sugimachi K (1994) S100 protein positive human breast carcinomas: an immunohistochemical study. *J Surg Oncol* **55**: 108–113

Messeri G, Tozzi P, Boddi V and Ciatto S (1983) Glucose-6-phosphate dehydrogenase activity and estrogen receptors in human breast cancer. *J Steroid Biochem* **19**: 1647–1650

Meyer I, Thompson J, Kiser E and Haven G (1980) Observation of a variant creatine kinase isoenzyme in sera and breast tumor cytosols. *Am J Clin Pathol* **74**: 332–336

Nanji A (1983) Serum creatine kinase isoenzymes: a review. *Muscle Nerve* **6**: 83–90

Nesland J, Memoli V, Holm R, Gould V and Johannessen J (1985) Breast carcinomas with neuroendocrine differentiation. *Ultrastruct Pathol* **8**: 225–240

Nesland J, Holm R and Johannessen J (1986*a*) A study of different markers for neuroendocrine differentiation in breast carcinomas. *Path Res Pract* **181**: 524–530

Nesland J, Holm R, Johannessen J and Gould V (1986*b*) Neurone-specific enolase immunostaining in the diagnosis of breast carcinomas with neuroendocrine differentiation. Its usefulness and limitations. *J Pathol* **148**: 35–43

Nesland J, Lundi S, Holm R and Johannessen J (1987) Electron microscopy and immunostaining of the normal breast and its benign lesions: a search for neuroendocrine cells. *Histol Histopath* **2**: 73–77

Nesland J, Holm R, Johannessen J and Gould V (1988) Neuroendocrine differentiation in breast lesions. *Path Res Pract* **183**: 214–221

Nesland J, Ottestad L, Heikilla R, Holm R, Tveit K and Borresen A-L (1991*a*) C-*erb*B-2 protein and neuroendocrine expression in breast carcinomas. *Anticancer Res* **11**: 161–168

Nesland J, Ottestad L, Borresen A-L, Tvedt K, Holm R, Heikkilä R and Tveit K (1991*b*) The c-*erb*B-2 protein in primary and metastatic breast carcinomas. *Ultrastruct Pathol* **15**: 281–289

Omenn G and Cheung C-Y (1974) Phosphoglycerate mutase isozyme marker for tissue differentiation in man. *Am J Hum Genet* **26**: 393–399

Omenn G and Hermodson M (1975) Human phosphoglycerate mutase: isozyme marker for muscle differentiation and for neoplasia. In *Isozymes* Vol. 3 (Markert Cl ed) pp. 1005–1019. Academic Press: New York

Osborne K (1985) Heterogeneity in hormone receptor status in primary and metastatic breast cancer. *Semin Oncol* **12**: 317–326

Pahlman S, Esscher T and Nilsson K (1986) Expression of $\gamma$-subunit of enolase, neuron-specific enolase, in human non-neuroendocrine tumors and derived cell lines. *Lab Invest* **54**: 554–560

Pertschuk L, Eisenberg K and Carter A (1985) Heterogeneity of estrogen binding sites in breast cancer: morphologic demonstration and relationship to endocrine response. *Breast Cancer Res Treat* **5**: 137–147

Rapoport S (1968) The regulation of glycolysis in mammalian erythrocytes. *Essays Biochem* **4**: 69–103

Reeve J, Stewart J, Watson D, Wulfrank D, Twentyman P and Bleehen N (1986) Neuron specific enolase expression in carcinoma of the lung. *Br J Cancer* **53**: 519–528

Royds J, Taylor C and Timperley W (1985) Enolase isoenzymes as diagnostic markers. *Neophatol Appl Neurobiol* **11**: 1–16

Scambia G, Natoli V, Panici P, Sica G and Mancuso S (1986*a*) *J Cancer Res Clin Oncol* **112**: 29–32

Scambia G, Panici P, Sica G, Natoli V, Caruso A and Mancuso S (1986*b*) Creatine kinase activity and steroidal hormone receptors in primary breast cancer. *Ann N Y Acad Sci* **464**: 511–513

Scambia G, Santeunasio G, Panici P, Iacobelli S and Mancuso S (1988) Immunochemical localization of creatine kinase BB in primary breast cancer: correlation with estrogen receptor content. *J Cancer Res Clin Oncol* **114**: 101–104

Schmechel D (1985) $\gamma$-Subunit of the glycolitic enzyme enolase: nonspecific or neuron specific? *Lab Invest* **52**: 239–242

Schmechel D, Marangos P and Brightman M (1978) Neuron-specific enolase is a molecular marker for peripheral and central neuroendocrine cells. *Nature* **276**: 834–836

Scopsi L, Andreola S, Pilotti S, Testori A, Baldini M, Leoni F, Lombardi L, Hutton J, Shimizu F, Rosa P et al (1992) Argyrophilia and granin (chromogranin/secretogranin) expression in female breast carcinomas. Their relationship to survival and other disease parameters. *Am J Surg Pathol* **16**: 561–576

Taylor C, Royds J, Parsons M and Timperley W (1983) Diagnostic aspects of enolase isozymes. *Curr Topics Biol Med Res* **11**: 95–119

Tsung A (1983) Creatine kinase activity and isoenzyme pattern in various normal tissues and neoplasms. *Clin Chem* **29**: 2040–2043

Urdal P, Urdal K and Stromme J (1983) Cytoplasmic creatine kinase isoenzymes quantitated in tissue specimens obtained at surgery. *Clin Chem* **29**: 310–313

Vinores S, Bonnin J, Rubinstein L and Marangos P (1984) Immunohistochemical demonstration of neuron-specific enolase in neoplasms of the CNS and other tissues. *Arch Pathol Lab Med* **108**: 536–539

Wallimann T, Wyss M, Bridiczka D, Nicolay K and Eppenberger M (1992) Intracellular compartmentation, structure and function of creatine kinase isoenzymes in tissues with high and fluctuating energy demands: the ‘phosphocreatine circuit’ for cellular energy homeostais. *Biochem J* **281**: 21–40

Wilander E, Phalman S, Sällström J and Lindgrem A (1987) Neuron-specific enolase expression and neuroendocrine differentiation in carcinomas of the breast. *Arch Pathol Lab Med* **111**: 830–832.

Winstend S and Hopps J (1985) Enzyme studies in breast tumor cytosols. *Clin Chem* **31**: 986

Wold F (1971) Enolase. In: *The Enzymes*, Vol V, Boyer P D (ed), pp. 499–538. Academic Press: New York

Wold L, Chin-Yang L and Homburger H (1981) Localization of the B and M polypeptide subunits of creatine kinase in normal and neoplastic human tissues by an immunoperoxidase technic. *Am J Clin Pathol* **75**: 327–332

Wyss M, Smeitink J, Wevers R and Wallimann T (1992) Mitochondrial creatine kinase: a key enzyme of aerobic energy metabolism. *Biochim Biophys Acta* **1102**: 119–166

Zarghami N, Yu H, Diamandis E and Sutherland D (1995) Quantification of creatine kinase BB isoenzyme in tumor cytosols and serum with an ultrasensitive time-resolved immunofluorometric technique. *Clin Biochem* **28**: 243–253

Zarghami N, Giai M, Yu H, Roagna R, Ponzone R, Katsaros D, Sismondi P and Diamandis E (1996) Ceratine kinase BB isoenzyme levels in tumour cytosols and survival of breast cancer patients. *Br J Cancer* **73**: 386–390

Zeltzer P, Schneider S, Marangos P and Zweig M (1986) Differential expression of neural isozymes by human medulloblastomas and gliomas and neuroectodermal cell lines. *J Natl Cancer Inst* **77**: 625–631.

*© 2000 Cancer Research Campaign*
